# Langerhans cell histiocytosis: Current concepts in dentistry and case report

**DOI:** 10.4317/jced.52498

**Published:** 2016-02-01

**Authors:** Efraín Ramos-Gutiérrez, Francisco Alejo-González, Socorro Ruiz-Rodríguez, José-Arturo Garrocho-Rangel, Amaury Pozos-Guillén

**Affiliations:** 1 DDS, Resident, Pediatric Dentistry Postgraduate Program, Faculty of Dentistry, San Luis Potosi University, SLP, Mexico; 2 MD, MS, Associate Professor, Pediatric Dentistry Postgraduate Program, Faculty of Dentistry, San Luis Potosi University, SLP, Mexico; 3 DDS, MS, Chairman, Pediatric Dentistry Postgraduate Program, Faculty of Dentistry, San Luis Potosi University, SLP, Mexico; 4 DDS, MS, PhD, Associate Professor, Pediatric Dentistry Postgraduate Program, Faculty of Dentistry, San Luis Potosi University, SLP, Mexico; 5 DDS, MS, PhD, Associate Professor, Pediatric Dentistry Postgraduate Program, Faculty of Dentistry, San Luis Potosi University, SLP, Mexico

## Abstract

Langerhans cell histiocytosis (LCH), which is a rare granulomatous pediatric disease of unknown etiology, is characterized by the idiopathic proliferation and accumulation of abnormal and clonal Langerhans cells or their marrow precursors, resulting in localized, solitary or multiple destructive lesions. These lesions are most commonly eosinophilic granuloma, which are found in craniofacial bone structures such as the skull and mandible, skin and other organs. In children, the disease has a variable initial presentation, and the clinical course, prognosis and survival are unpredictable. The aims of this report were to present an LCH case in a girl aged 2 years, 8 months and her clinicopathological features, to describe the bucodental management provided, and to discuss special dental considerations of this disease.

** Key words:**Children, dental management, histiocytosis, Langerhans cells.

## Introduction

Langerhans cell histiocytosis (LCH), previously named histiocytosis X, is an uncommon hematological disease that predominantly affects infants and young children. LCH is characterized by a proliferation, infiltration and accumulation of a specific histiocyte, namely, the pathological Langerhans cell, which is considered to be a special type of dendritic mononuclear cell, within a variety of tissues and organs (1,2). Such dendritic mononuclear cells are derived from the bone marrow and possess important immunologic functions, particularly in antigen development of T-lymphocytes (3,4). The monoclonal infiltration of Langerhans cells, together with other immune effector cells, causes localized destructive tissue lesions as a result of the cellular infiltration, which replaces bone and invades skin, mucosa and internal organs (5,6).

The disease was firstly described in 1865 by Dr. Thomas Smith, who reported the case of a 4 years old child with impetigo and three large bone defects on the calvary. In 1868, the German physiologist Paul Langerhans observed several non-pigmented dendritic cells in the epidermis and mucous membrane, which he initially considered to be neuronal, and then corrected himself. Subsequently and independently of each other, Dr. Alfred Hand, Dr. Artur Schüller and Dr. Henry Christian described similar cases (7,8). In 1953, Dr. Louis Lichtenstein introduced the term ‘histiocytosis X,’ which characterized the disease as a proliferation of histiocytes of unclear etiology, and it was unknown whether it should be classified as a neoplastic, inflammatory or lipid disorder (9,10). In 1987, the Histiocyte Society was founded with the collaboration of several international research organizations; since then, the society has established diverse standards regarding the definition, classification and general management of LCH (11).

The pathogenesis of LCH remains unclear and poorly understood; however, according to Merglová *et al.*, various etiologic factors have been proposed, such as neonatal infections, a skipped vaccination, exposure to solvents, and thyroid diseases (10). Approximately 200 new cases of LCH are diagnosed each year in the US, whereas in Western Europe, it is estimated that LCH occurs at a rate of 2 to 5 cases per million per year, primarily in children under the age of 15 (the incidence peaks between 1 year and 4 years, although some cases have been reported in newborns); 80% are Caucasian, and boys are affected more than girls, with a reported male:female ratio ranging from 2:1 to 4.6:1. Approximately 99% of patients are diagnosed with type I or II variety. Its incidence is approximately 10% that of acute childhood leukemias (4,5,12).

-Search strategy

This review was based on a Medline-PubMed, Scopus, and Web of Knowledge searching, which was conducted to obtain papers published in English and Spanish languages, between June 1999 and December 2014, using the following MESH terms or key words, in different combinations: *“Langerhans”, “histiocytosis”, “children”, “dental management”*. We also conducted a ma-nual searching of relevant papers published in four major oral surgery journals (Journal of Craniomaxillofacial Surgery, Oral Maxillofacial Surgery, Oral Surgery Oral Medicine Oral Pathology Oral Radiology Endodontology, and Medicina Oral Patología Oral Cirugía Bucal), and three pediatric dentistry journals (Pediatric Dentistry, Journal of Clinical Pediatric Dentistry, International Journal of Paediatric Dentistry). Both electronic and manual searching yielded in total sixty-seven potentially relevant papers, based on the review of the corresponding abstracts. Then, following the full-text critical evaluation, thirty-three studies were finally included in the review.

## Case Report

A Mexican girl aged 2 years, 8 months presented to the Dental Clinic, referred by a general dental practitioner, with a chief complaint of “strongly decayed, painful teeth and gum bleeding”. Previously to the examination a written informed consent was obtained. The parents did not report craniofacial trauma, medication use, environmental allergies, or previous surgeries. From the age of 1 ½ years, the patient manifested recurrent episodes of skin erythema and rash, otitis media, anemia and fever. After complete medical and dental evaluation, a skin biopsy was collected and an immunohistochemical analysis was performed, which demonstrated positive results for CD 1a (d10, Labvision) and for S-100 (Policlonal, DAKO).

**Figure 1:**
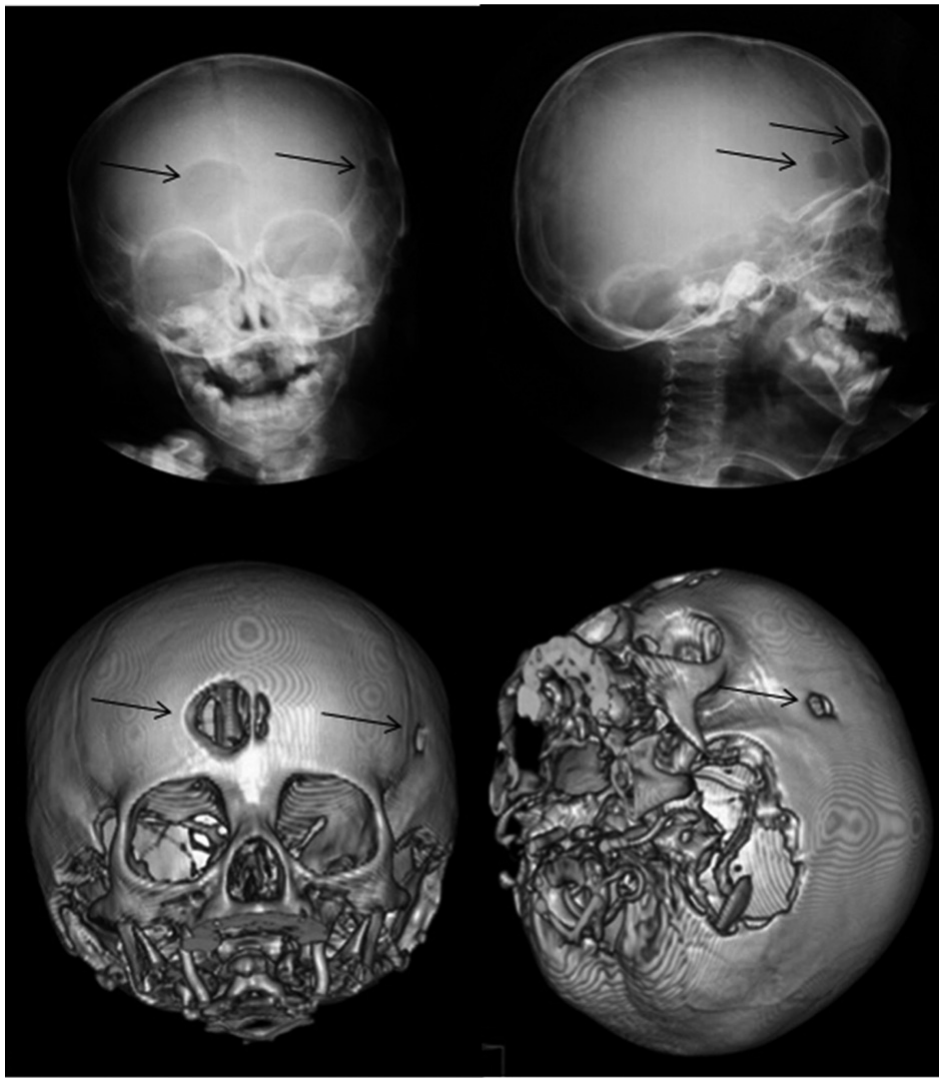
Frontal and lateral radiograph of the skull showing a punched-out lesion in the frontal and temporal bone (arrows). A 3-dimensional CAD view of cranial bones of the same patient demonstrating osteolytic lesions (arrows).

Subsequently, an imaging survey was performed: frontal (anteroposterior) and lateral cranial x-rays, and a 3-dimensional CAD view of cranial bones. The skull x-rays and 3-dimensional model exhibited two well-defined bone lesions with demarcated borders: the first lesion appeared in the right-central portion of the frontal bone, approximately 1 cm wide, ovoid form and divided into two cavities by a thin bone ridge; and the second lesion appeared in the left temporal bone, 3 mm wide, circular form, 1 cm from the orbit in the horizontal plane (Fig. 1). Additionally, the patient exhibited hepatomegaly, splenomegaly, and bone marrow cell infiltration. Based on the data collected, the child was diagnosed with LCH.

**Figure 2:**
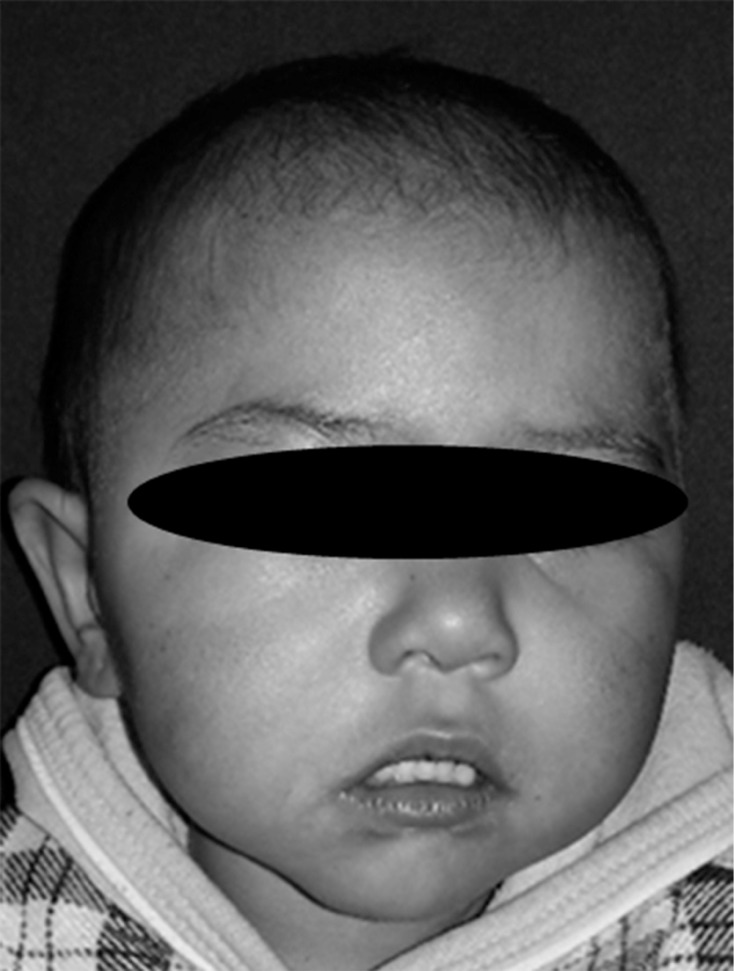
Extraoral view.

At the time of the first dental examination in our clinic, the patient was receiving chemotherapy, and did not report any symptoms or discomfort related to the disease or treatment. When she was physically examined, the patient exhibited good general health status and a markedly uncooperative behavior. Except for her thinning hair, there was no evidence of abnormal extraoral signs (Fig. 2).

The patient’s oral cavity exhibited an almost completely erupted deciduous dentition, with mild incisor crowding, poor hygiene, excessive plaque accumulation, and halitosis; none of the teeth demonstrated abnormal mobility. Soft tissue did not exhibit any abnormality. Deep carious cavities were present in both lower and left upper first molars, the right upper second molar and canine, and root remnants of the first right upper molar. The parents mentioned that she had moderate dental pain during eating. Approximately 1 year ago, they noted the presence of a soft “small bulge” in the palatal area, which resolved spontaneously in a few months.

**Figure 3:**
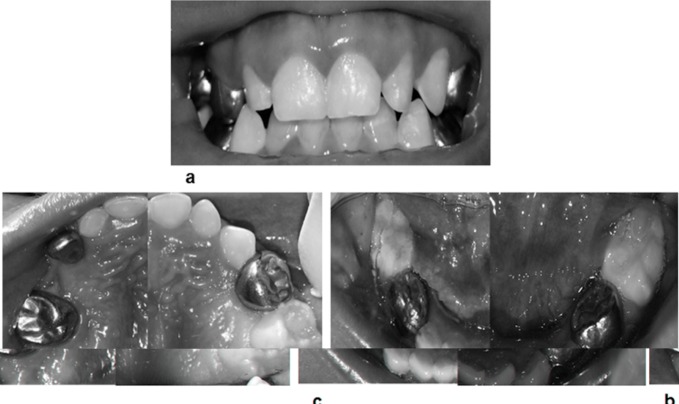
a) Frontal intraoral view; b) Intraoral view of maxillary arch; c) Intraoral view of mandibular arch.

Considering the patient´s age, her poor cooperation, and chemotherapy, we decided to conduct the entire oral rehabilitation under general anesthesia, once she was considered to be systemically stable. The parents fully understood and agreed with the treatment plan, and they provided written informed consent. The treatment consisted of performing five pulpotomies, which were then restored with stainless steel preformed crowns; the extraction of root remnants, and the subsequent positioning of a band loop space maintainer. Additionally, pit and fissure sealants were placed on both partially erupted lower second molars (Fig. 3). The process of general anesthesia followed the strict guidelines recommended by the American Academy of Pediatric Dentistry, in terms of care to minimize risks for the child. There were no adverse local or systemic events during and after the treatment. At subsequent appointments, the parents received instructions regarding their daughter’s oral hygiene and nutritional guidance. Future control reviews were carefully scheduled.

Patients’ parents accepted that their correspondent case report were submitted to a scientific dental journal, by means of a given signed written consent, after authors clearly and completely informed them about the possible benefits to dental science, in terms of knowledge and generation of research hypothesis for future higher quality studies.

## Discussion

Traditionally, the International Histiocyte Society has classified LCH into three different types, based on the different clinical manifestations: Type I, Eosinophilic granuloma (chronic focal LCH), which only involves a solitary or multiple bone lesions; Type II, Hans-Schüller-Christian disease (chronic disseminated LCH), which is characterized by the classical triad: lytic bone lesions, exophthalmos, and diabetes insipidus; and Type III, Abt-Letterer-Siwe disease (acute disseminated LCH), considered as the malignant form of LCH (2,8,12). However, because clinical findings are widely manifested, the disease has also been classified into unifocal and multifocal forms according to the extent of its spread throughout the body. The unifocal form presents only one destructive lesion in an organ, affecting more frequently tissues such as bones or skin (80% of cases), liver, spleen, brain, and lymph nodes (6).

The type of osteolytic localized lesion most common in pediatric LCH is the eosinophilic granuloma. Although the disease can cause lesions in any bone, previous observational studies have shown that head and neck are the most common anatomical areas where LCH bone lesions are present, with an incidence rate of 65% to 90%, including the skull (particularly the calvarium, temporal bones, and the sella turcica), mandible or maxilla (5,13). Quaishi *et al.* (14) performed a study (between 1959 to 1993) that included 73 British pediatric patients with LCH and found 67% of patients with head and neck involvement, primarily manifested as localized bony lesions in the skull. Similarly, in a 10-year retrospective study on patients under 14 years old with LCH, Cochrane *et al.* (15) reported that 57% of these patients exhibited localized osteolytic lesions, most frequently (67%) in the head and neck regions; the remaining 43% manifested the multifocal presentation of the disease. In another retrospective study (with an 18-year follow-up period) with 22 pediatric patients conducted by Buchmann *et al.* (16), 17 patients had head and neck involvement, and in 14 patients these lesions were the primary bodily manifestation. Artzi *et al.* (17) mentioned that LCH patients with head and neck lesions manifested characteristic signs and symptoms of the periodontal disease. Thus, the evidence of destructive periodontitis, abnormal mobility or tooth loss as the initial child’s complaints during the primary dentition stage may be indicative of a systemic condition, such as LCH; for purposes of confirming the diagnosis using immunofluorescence tests, it is strongly recommended that the dentist collect sufficient gingival biopsy material, with or without tooth extraction. In 2009, from their case-and-control research, Alexiou *et al.* (18) reported 22 cases of children with skull solitary eosinophilic granuloma, who were predominantly male, had a mean age of 7.5 years, and in whom the frontal bone was the most commonly involved.

Radiographically, the bone lesions usually appear as well defined central destructive-related radiolucencies; in the skull, these lesions develop in the diploic space, with sharply circumscribed, scalloped or confluent edges, or show a “button sequestrum” (13,19). Flat and long bones, ribs, pelvis and shoulder blade are also affected; these type of lesions may cause pain and adjacent soft-tissue swelling, especially during exercise. In the multifocal form, more than one organ is involved and may include other anomalies, such as hepatosplenomegaly, pulmonary disease, and cervical lymphadenopathy; occasionally, the disease is related to otitis, deafness, leukocytosis, fever, and endocrine gland and central nervous system involvement (8,10).

The initial LCH manifestations are often skin and oral changes. Seborrheic skin lesions or atopic dermatitis are observed in 38% of pediatric patients. In addition, the oral cavity may be the only affected area and the incidence of oral lesions is as frequent as 77%. Oral lesions may be the initial clinical signs or complaints in all forms of LCH, and 10-20% of them are considered to be nonspecific for the disease (6,19,20). Gingivitis with hyperplasia, bleeding, recession and necrosis, mucosal ulcerations and destruction of the alveolar bone and periodontal support, impaired healing, abnormal primary dentition mobility (‘floating teeth’), intraoral mass, halitosis, and odontalgia are common findings in pediatric patients (12,21). Furthermore, lytic bone lesions in the jaw are predominant (5% to 10% of cases), primarily in the posterior region (distal to the canines) and ramus of the mandible, occasionally accompanied by facial pain and swelling, and in severe cases, pathological jaw fractures; they may disturb the tooth-germ development and cause enamel hypoplasia of the permanent teeth (3,22,23). Other facial, oral abnormalities and bone lesions are described in  Table 1.

**Table 1:**
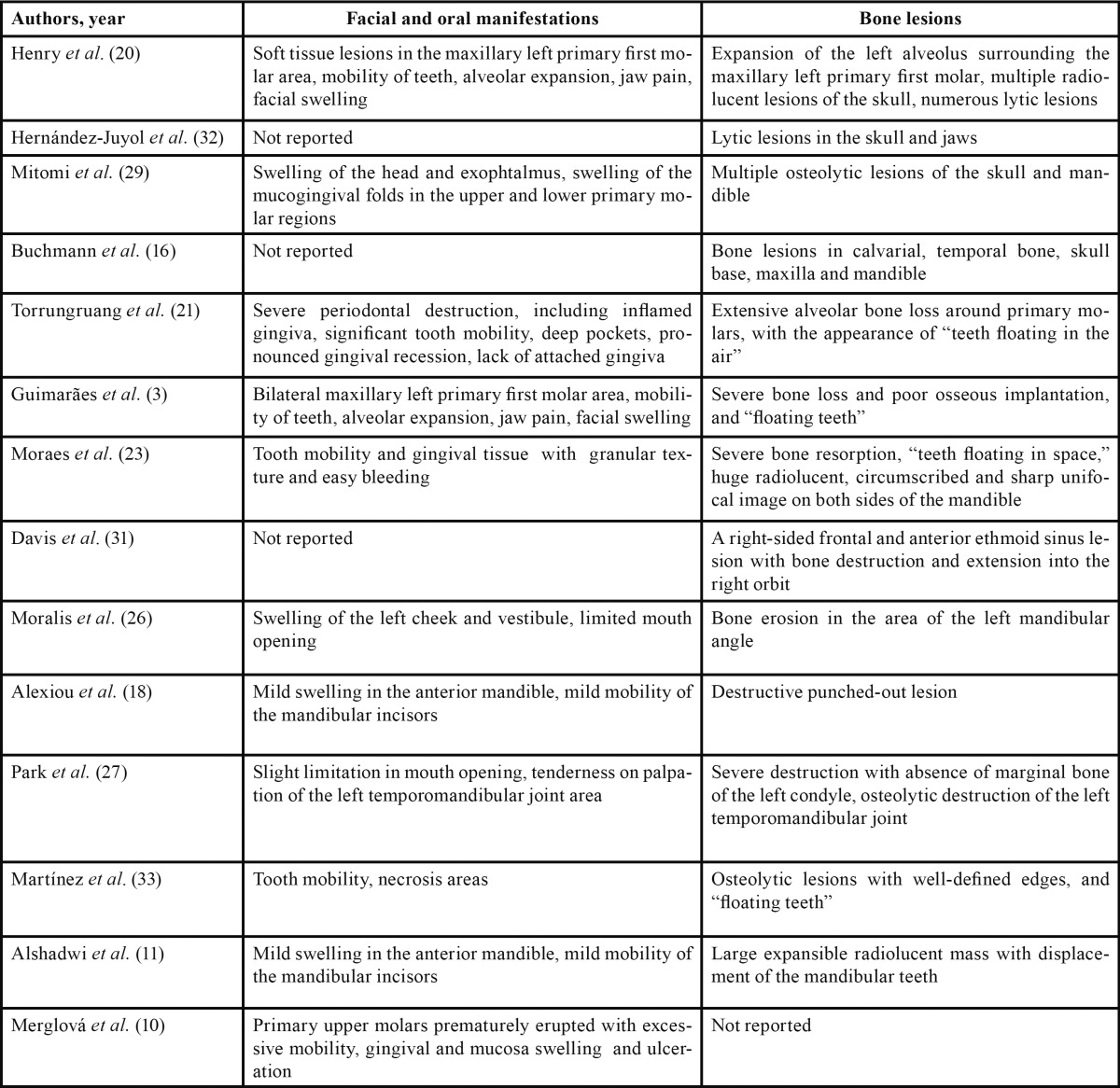
Features associated with Langerhans cell histiocytosis.

Through a clinical, imagenological and histopathological examination, a definitive diagnosis of LCH can be established. Certain immunohistochemical findings are considered to be characteristic of the disorder, such as high levels of salivary interleukin-Iβ and prostaglandin E2, positive staining for S100 protein, Langerin (CD207) monoclonal antibody, and identification of specific cytoplasmic organelles known as Birbeck’s granules, or the CD1 antigen on the surface of the Langerhans cell (10,20,24). Other systemic abnormalities with similar clinical manifestations include leukemia, cyclic neutropenia, hypophosphatasia, fibrous dysplasia, Papillon-Lefèvre syndrome, agranulocytosis, malignant neoplasms, sarcoidosis, and giant cell disorders (3,5). In dentistry, the primary differential diagnoses include advanced periodontal disease, diabetes-associated or a periapical process of dental or periodontal origin (3).

Although a specific therapeutic protocol for LCH does not exist, the Histiocyte Society published in 2009 the Evaluation and Treatment Guidelines based on current best practices in the treatment of LCH (25). In general, available treatment options include combinations of surgical removal of localized bone lesions, chemotherapy and radiation (6). Diverse agents (immunosuppressant agents, immune modulators, and cytostatic agents) have been employed, such as corticosteroids, antibiotics, prednisonevinblastine combination, vincristine sulphate, indomethacin, methotrexate, cyclophosphamide, indomethacin, adrenocorticotropic hormone (ACTH), etoposide, and 6-mercaptopurine (26,27); or therapeutic procedures, such as interferon or cyclosporine-A, bone marrow transplant, monoclonal anti-CD 1a antibody therapy, and gene transfer into hemopoietic progenitor or stem cells (26). Treatment of the disease depends primarily on a careful multidisciplinary evaluation and correct diagnosis. According to the Histiocyte Society, the three points of confidence for a precise diagnosis of LCH in children are as follows: 1) clinical and radiographic oral characteristics, for example, displaced teeth and developing permanent tooth follicles with altered eruption patterns or dentoalveolar growth; 2) two or more positive stains for adenosinetriphosphatase, S-100 protein, alpha-D-mannosidase, or presence of peanut lectin; and 3) detection of Birbeck granules and CD 1a antigen (T6) on the surface of Langerhans cells. Once the diagnosis is confirmed, the treatment team must perform skeletal and visceral system assessments to verify the presence of other lesions in the rest of the patient’s body (6,10).

In general, the long-term craniofacial management of patients with LCH starts with a review of the natural history of the specific disorder, location, and therapeutic scheme currently administered (22,27). Bartnick *et al.* (28) recommended adhering to the staging of disease to determine the appropriate treatment plan and prognosis as follows: Stage I, a unique lesion; stage II, multiple lesions; and stage III, concurrent lesions in the craniofacial region and other organs. Additionally, the clinicians must consider whether the lesions are located in bone or soft tissues or within the internal organs. Thus, in stages I and II, when only the bone or only the soft tissues are involved, the treatment is solely surgical. In stage III, a combination of chemotherapy, radiation (in recommended doses of 6 Grays to 15 Grays), and pharmacotherapy (1,26). However, in young pediatric patients, especially in those under 5 to 6 years of age, radiation therapy may seriously affect the developing teeth and craniofacial bones, as well as adversely affect the intellectual development; hence, radiation therapy is not suggested as the first-choice treatment in young children. Additionally, in LCH stage III cases, there is a long-term 1-5% risk of secondary malignancy, such as lymphoma, acute leukemia or solid tumors (10). Therefore, a more conservative drug therapy is preferred, for instance, intralesional corticosteroids (26); however, this approach may occasionally delay the root development of those teeth close to the osteolytic lesion (29).

The different available dental or craniofacial surgical treatment options for children with LCH have several aims, one of the most important being to provide not only functional results but also an esthetic orofacial appearance to prevent likely psychological sequelae and thus improve the patients’ quality of life (20,23). A common dental intervention is the extraction of primary teeth with severe mobility or in cases of osteolytic lesions surrounding the root apex; afterwards, it will be necessary to attempt the recovery of subsequent lost vertical dimension (3). Another important treatment aim related to the dental occlusion is to avoid any likely interference during the permanent teeth eruption (23).

The prognosis of LCH depends on three specific indicators (10,20): 1) Age: children under two years generally have disseminated disease and a poorer prognosis; 2)Number of sites involved: multifocal disease (especially when spleen, lungs, liver, or the hematopoietic system are infiltrated) indicates a poorer prognosis. In children under 2 years of age with multifocal dysfunction involvement, the disorder is considered to be life-threatening, with a mortality rate of approximately 60%; and 3) Organ dysfunction, when present, results in a poorer prognosis. On the other hand, LCH stages I and II have shown cure rates up to 80%, whereas in stage III, the rate decreases between 2% and 25%; the overall 5-year survival rate for LCH is approximately 92% (1,11). Although cases of spontaneous remission over a period of many months or years have been observed, several cases have a long-lasting course with remissions, recurrences or disseminations. Lau *et al.* (30) reported an incidence of permanent sequel in 29% of surviving patients, such as diabetes insipidus, exophtalmus, orthopedic anomalies, hearing loss, and neurological abnormalities; in several cases, such sequelae manifested up to 10 years after the initial diagnosis of LCH. It has been reported that the disorder dissemination (unifocal to multifocal) is most frequent in children under 5 years of age, within the first 6 months after the initial diagnosis (6,23).

The long-term dentofacial development of children treated for LCH has not been fully reported; however, dentists and parents should be aware of the different chemoradiation sequelae, primarily in children under 5 and 6 years old; commonly reported anomalies include the following: localized enamel defects, tooth agenesia, altered dentoalveolar growth, adverse intellectual effects, and second tumor development (3,20).

As mentioned above, although not always pathognomic, oral findings in hard and soft tissues, such as local pain, swelling and ulcerations, may be the first or only clinical pathological manifestations of Langerhans cell histiocytosis. These oral changes should be of special interest for dentists because they may play a critical role in detecting LCH, and they are usually the first clinicians to examine the child’s oral cavity and participate in the diagnosis and care processes of the disease. An early diagnosis and appropriate referral are significant because the disease is, for the most part, fatal in severe cases. In addition to those clinical findings, it is essential to perform an image (radiographs and computer views) examination from the skull in suspected patients; however, it is difficult to make a diagnosis without confirmatory immunohistochemical tests, such as a positive CD 1a or S-100, because LCH bone lesions may be easily confounded with a malignant process, which results in unnecessary and likely aggressive management (18,27). In contrast, a more serious anomaly may be overlooked; for instance, Davis *et al.* (31) reported a case of an 11-year-old male, in whom a lytic lesion located in the front-orbital and anterior ethmoid sinus bones was mimicking an aggressive osteomyelitis (Pott’s puffy tumor). Therefore, the treatment team must make a careful and well-defined diagnosis to provide an adequate therapeutic approach, maintaining the most optimal quality of life, with the fewest side-effects.

## Conclusions

Dentists may play in important role in the multidisciplinary treatment team that manages children with LCH, by performing careful early oral examinations and long-term controls during the follow-up period of the disease, for detecting associated oral findings, which may be the initial and only manifestations of the disorder. Therefore, clinicians must be always vigilant to discern dental and mucosal abnormalities in their patients with suspected LCH.
